# Simulation centres in German hospitals and their organisational aspects: Expert survey on drivers and obstacles

**DOI:** 10.3205/zma001186

**Published:** 2018-08-15

**Authors:** Tobias Rampel, Benedict Gross, Alexandra Zech, Stephan Prückner

**Affiliations:** 1 Klinikum der Universität München, Institut für Notfallmedizin und Medizinmanagement – INM, München, Germany; 2 University for Professional Studies, Berlin, Germany

**Keywords:** Patient Simulation, Simulation Training, Economic Factors

## Abstract

**Background: **Simulation trainings offer the opportunity to replicate parts of or entire processes of medical care in a controlled environment. Some hospitals operate simulation centres dedicated to training their staff. Which organisational factors support or impede the implementation of such units in hospitals?

**Methods:** In an exploratory survey experts and decision makers in German hospitals were interviewed. The answers were evaluated qualitatively in order to identify patterns in argumentation. Among the eleven participants were practitioners heading simulation centres (n=4), managers or executives in charge of finances (n=2), medical directors or head physicians with disciplinary responsibility for medical personnel (n=3), and researchers who deal with simulation trainings in medicine (n=2). The interview partners were guaranteed confidentiality in order to allow for them to speak freely about the obstacles and weaknesses in their respective organisations.

**Results: **The interviewees showed a very heterogeneous picture of the simulation trainings in their respective hospitals in terms of content as well as target groups. Asked about purpose and benefit of simulation centres, subgroups of the interviewees gave various arguments. Financing is comprised of subsidies, fees from external participants, and of vocational training budgets. Several obstacles for the implementation were mentioned such as insufficient error culture, deficient interaction of quality and risk management, high equipment costs, or staffing levels that are too low to schedule entire teams for vocational trainings.

**Conclusions:** Patterns in argumentation could be identified that support the implementation and operation of simulation centres in hospitals. Yet strikingly enough there were no coherent arguments i.e. there was no uniform reasoning of aim and purpose of simulation centres. Furthermore, the survey indicated the need to approach quality- and risk management more holistically wherefore simulation centres could offer laboratory environments. All in all, the survey indicates that the assessment of success and economic benefits of simulation centres haven’t yet been examined thoroughly.

## Zusammenfassung

**Hintergrund: **Simulationstrainings bieten die Möglichkeit, medizinische Versorgungsabläufe in kontrollierter Umgebung teilweise oder vollständig nachzubilden. Einige Krankenhäuser betreiben eigene Simulationszentren um ihr Personal zu trainieren. Welche Faktoren in der Organisation beeinflussen die Einführung solcher Zentren positiv oder negativ?

**Methoden: **In einer explorativen Umfrage wurden Entscheidungsträger deutscher Krankenhäuser interviewt. Die Antworten wurden qualitativ ausgewertet um Muster in den Ausführungen zu identifizieren. Unter den 11 Teilnehmern befanden sich Praktiker, die Simulationszentren leiten (n=4), Manager bzw. Vorstände, die in der Führungsebene von Krankenhäusern für die Finanzierung verantwortlich sind (n=2), Ärztliche Leiter bzw. Chefärzte mit disziplinarischer Verantwortung für medizinisches Personal (n=3) und Forscher, die sich mit Simulationstrainings in der Medizin beschäftigen (n=2). Den Interviewpartnern wurde Vertraulichkeit ihrer Aussagen zugesichert, um auch Hindernisse und Schwächen ihrer jeweiligen Organisation frei ansprechen zu können

**Ergebnisse:** Die Befragung zeichnete ein heterogenes Bild was die Angebote von Simulationstrainings in den jeweiligen Häusern angeht, sowohl hinsichtlich des Inhalts, als auch der Zielgruppen. Befragt zu Zweck und Nutzen von Simulationszentren führten die Subgruppen der Interviews sehr unterschiedliche Argumente an. Die Finanzierung erfolgt durch Quersubventionierung, Gebühren externer Kursteilnehmer und aus Fort- und Weiterbildungsbudgets. Es wurden mehrere Hindernisse der Einführung berichtet, etwa unzureichende Fehlerkultur, mangelhaftes Zusammenspiel zwischen Qualitäts- und Risikomanagement, hohe Anschaffungskosten oder zu geringe Personaldecken, um ganze Teams für Schulungen freizustellen.

**Schlussfolgerungen:** Es konnten Argumentationsmuster identifiziert werden, die für Einführung und den Betrieb von Simulationszentren in Krankenhäusern sprechen. Auffallend ist jedoch, dass die Argumentationen der interviewten Gruppen nicht kohärent zueinander waren, beispielsweise gab es keine einheitliche Begründung von Zweck und Nutzen von Simulationszentren. Weiterhin ergab die Befragung Hinweise auf eine Notwendigkeit, die Bereiche Qualitäts- und Risikomanagement ganzheitlich zu betrachten. Simulationszentren können einen Beitrag als Übungsumgebung dazu liefern. Insgesamt ergab die Umfrage, dass die Erfolgsmessung und der wirtschaftliche Nutzenbeweis von Simulationszentren bisher nur unzureichend betrachtet zu sein scheinen.

## Introduction

The high number of avoidable adverse events in medicine is addressed and discussed extensively [1], [2], [3], [4]. A known fact is that – regardless of actual frequency – human factors have an important role in the occurrence of preventable incidents [5]. Simulation trainings can contribute to the prevention and reduction thereof [6]. The understanding of simulation in the medical context is broadly formulated “an educational technique that allows interactive, and at times immersive, activity by recreating all or part of a clinical experience without exposing patients to the associated risks” [7]. The need for simulation trainings seems to increase with the rising complexity in medical treatment and a growing degree of mechanisation, but also an increased awareness for patient safety [8]. 

Furthermore, expert societies prominently promote simulation as a training method. For instance, the American Heart Association (AHA) emphasizes in its guidelines 2015: “There is substantial evidence to suggest that mastery learning is the key to skill retention and the prevention of rapid decay in skills and knowledge after simulation-based learning.” [9]. In contrast to this demand a 2009 survey suggests that the reality of application of this training method lacks behind in Germany: only about 67% of the surveyed hospitals offered resuscitation training and only 55% thereof more often than once a year [10]. Whereas the optimal frequency of life support trainings is still unclear, it is suggested that frequent manikin-based refresher training may save costs and reduce the total time for retraining [11]. Looking at manikin-based resuscitation training as a simple version of simulation trainings these numbers could indicate very limited prevalence. Another current survey of German anaesthesiology departments conducting simulation trainings found that to have nationwide trainings the capacities must be increased decidedly [12]. On average nursing staff only get to partake in such trainings every six years.

For the purpose of this survey we understand simulation centres as specific organisational units of hospitals, equipped with staff and material to provide simulation-based trainings to healthcare staff. In general, such simulation centres do not only focus on trainings for technical skills of patient treatment but also expand their activities in team skills and communication trainings.

Why are there no nationwide simulation trainings and centres in medicine the way they exist in aviation? One reason could be that impact and economic success have not been proven empirically so far though the limitations and deficits of traditional vocational trainings are known [13]. The economic benefit of simulation trainings in particular has almost never been investigated at all; only scattered research is available on isolated and mostly technical aspects of simulation trainings such as the application of central venous catheters [14]. This evaluation found that simulation-based education reduced infection and saved consequential costs in an Intensive Care Unit.

To find reasoning and arguments supporting the implementation and use of simulation centres in German hospitals and to identify successful examples from practice we inquired experts and executives who either influenced or oversaw the implementation of such a unit.

## Method

Since the simulation expert community in Germany is manageable yet heterogeneous we chose an exploratory study design. The aim was to identify certain arguments and argumentation patterns with the use of qualitative methods. To do so, partly structured interviews were conducted. The interview outline consisted of 10 question blocks which were divided into three fields:

1. Descriptive data on hospital size as well as mode, timeframe, and extent of the offered simulation trainings.
2. Motivation for implementation of simulation trainings, specifically regarding economic considerations.
3. Identification of use, administrative figures, and interface of quality- and risk management.

The results from the third part of the survey were extremely manifold and can’t be reproduced entirely in this article. Noticeable aspects are summarized in successful examples from practice.

The research project has been proposed to and was approved upon review by the Berlin University for Professional Studies.

The survey participants were identified by analysis of literature, conference programmes, and internet research. Inclusion criteria were the functional or financial responsibility for the operation of simulation centres in a German hospital or research and publications related to simulation in medicine.

The identified experts were contacted via E-Mail with an enclosed letter explaining aims and methods of the study as well as the specific modality of the interview. To increase trustworthiness of the request and to minimise possible nonresponse bias, the letter was on official letterhead and signed by two of the researchers supervising this project (SP, BG). Recipients had to actively reply to our interview request and agree upon date and time for a call thus consenting their participation. All requested experts have been reminded once if they did not reply to the first E-Mail after two weeks. From 24 requests we received 11 responses and every response resulted in an interview, those who did not respond gave no reasons for refusal. After participants covering all fields of interest to our survey have been recruited, we passed on a second reminder.

Each interview was conducted along a semi-structured guideline (see attachment 1 ) and lasted from 25 to 45 minutes. Survey period was June to November 2015. Participants were guaranteed full anonymity at the beginning of the interview in order to allow them to speak freely about the obstacles and weaknesses in their respective organisation. Before the interview started all participants were asked for approval to record it. The interviews were conducted by one researcher (TR), audiotapes were deleted after transcription. To maintain confidentiality results are presented in groups or in a way that no information could be traced back to an individual participant. The successful examples from practice in this article do not contain any information that would allow for an individual person or hospital to be identified.

Following the phone calls all recordings were transcribed and evaluated by qualitative means [15]: In a first cycle of “descriptive coding” a detailed inventory of the transcripts was compiled by assigning labels summarizing the passages of the interviews. In a second cycle the codes were condensed under “pattern codes” grouping the information on a higher level to allow drawing conclusions. 

The interviewees were divided into four groups as to compare arguments and statements:

1. Practitioners in charge of running simulation centres
2. Managers on executive levels in hospitals in charge of financing, e.g. executive board members or Chief Financial Officer (CFO)
3. Medical directors or head physicians with disciplinary responsibility for medical personnel
4. Researchers dealing with simulation trainings in medicine

The participants (n=11) consisted of 4 practitioners, 2 managers, 3 medical directors, and 2 researchers (see Table 1 (Tab. 1)). Both researchers do not have a simulation centre in their respective institutions. One of them consulted the establishment of various simulation centres. The majority of interview partners were physicians by education and have been promoted to management positions during their career. Three of the practitioners in charge of running simulation centres were physicians with a specialisation in anaesthesiology. The participants represent individual hospitals as well as networks of hospitals. In total our interview partners draw insight from a sample of hospitals encompassing about 9,2% of all beds nationwide.

## Results

The hospitals in question displayed no uniform picture with regards to simulation trainings. They mostly focussed on few selected fields or disciplines. While regular resuscitation trainings to varied extents were compulsory for the personnel of some houses (e.g. ACLS-concepts for anaesthesiology, Intensive medicine, and ICU as well as BLS-concepts for wards) interview partners from other hospitals commented on the lack of common standards and only trained certain departments regularly. 

The integration and participation of assistant and nursing personnel was being handled very inconsistently as well. Some houses made a point of training interdisciplinary teams while others paid only very little attention to it. The regularity of trainings and the commitment towards employees was influenced by external conditions such as the demand for the maintenance of certifications. 

The following gives statements of the interview partners arranged by topics. The number of mentions is given with every statement. 

### Target group of simulation trainings in hospitals

The hospitals in question almost unanimously catered their simulation trainings to certain departments only, such as ICU (n=4), trauma room (n=1), anaesthesiology (n=7), ER (n=2), EMS (n=3), catheter laboratory (n=1), and obstetrics (n=2) (multiple mentions were possible). Thus, predominantly the medical specializations anaesthesiology, paediatrics, obstetrics, and cardiology came in contact with such trainings.

#### Purpose of simulation trainings

The interviews’ evaluation showed that the groups use very different arguments, justifying the implementation or the operation of simulation centres. 

The practitioners argued from the view of the employee and mentioned better education (n=2), the training of emergency incidents (n=3), and the improvement of emergency management (n=2). An improvement in quality would also be part of a functioning inner-clinical risk management system (n=2). 

From the medical directors’ point of view the advantages were to be seen in an improvement of education (n=2) as well as in the employees‘ feedback (n=1) thus a faster orientation (n=2) and a more efficient deployment of employees (n=2). The chance for process improvements as part of a quality management were mentioned as well (n=1).

The researchers mentioned the benefit of modern technical equipment for educational purposes (n=1) and the increased efficiency due to good preparation and a steep learning curve in simulator trainings (n=1) as reasons for the implementation of a simulation centre. 

The members of hospital managements participating in the survey unanimously mentioned that simulation trainings were part of the effort to increase patient safety (n=2). Furthermore, they wanted to increase employee satisfaction (n=1) as well as the hospital‘s image (n=1). The focus on risk management (n=2) was obvious for these interview partners. The prevention of near-incidents (n=2) and the future handling of liability cases respectively insurance issues (n=2) were in the managers’ specific interest. In the end this also aimed at the hospitals’ profitability.

#### Financing of simulation centres

The interviewees’ simulation centres were mainly financed by cross-subsidization and fees from external training participants (n=5). Additionally, many houses had allocated budgets for vocational training for employees (n=7).

According to some of the interview partners the operation of a simulation centre offers advantages over the assignment of external service providers (n=2). 

Several of the considered hospitals conducted a make-or-buy analysis (n=5) to evaluate if a simulation centre should be implemented on their own premises or bought as an external service. Apart from lower overall costs one of the reasons for a simulation centre was a quick adaptation to internal specifications (n=2) as well as a better adjustment to individual needs (n=1). 

#### Controlling simulation centres through performance indicators

The interview covered questions regarding Key Performance Indicators (KPI). Whereas all interview partners mentioned training evaluations by participant questionnaires, none of them reported a full KPI system to monitor the simulation centre on an organisational level. However, various interview partners considered some performance indicators as probably related to the impact of simulation centres, such as patient satisfaction, staff satisfaction, labour turnover, evaluation results of resuscitations or trauma care and damage sum of medical malpractice. 

#### Problems in implementation of simulation centres

When asked about problems of the implementation of simulation centres, the interview partners mentioned differing factors. For one the leitmotif of risk management and the reality thereof were in stark contrast. The problems were seen in insufficient error culture (n=3) and the lack of interplay among quality- and risk management (n=4). Data from reporting systems such as Critical Incident Reporting Systems (CIRS) were not used sufficiently to support improvement. In addition to high costs of implementation and difficulties of financing thereof there are high costs caused by the absence of employees during trainings (n=4). In practice low staffing levels pose a problem, especially when team trainings should take place with given teams. Another issue mentioned was that the goals of the trainings weren’t achieved at all or only inadequately, because the contents of skill training and team training were taught in very short time which overburdened participants (n=2).

#### Examples of good practice

In the third part of the survey the interview partners were asked about reference figures with respect to the implementation of the simulation trainings. It transpired that the simulation trainings were evaluated concerning participants‘ satisfaction yet there seems to be no evaluation of the economic profitability. Thus, there was no information that could lead to a conclusion on economic profitability or efficiency monitoring of simulation centers.

During the interviews successful examples from practice were documented and are summarized below.

**Risk management as a financial incentive for management: **One of the interview partners stated his hospital defines patient safety as a company goal. The progression of critical incidents has direct influence on the variable salary component of the hospital’s management. This motivated the implementation of a structured risk and quality management as well as the establishment of an open error culture. One participant reported the simulation centre was used as an instrument to meet these figures by constant training of staff. 

**Adaptation of organisational structure:** One hospital put the simulation centre under the control of the medical director and assigned it to risk management. Closer organisational interplay should increase efficiency and the practical value of risk and quality management in this case. The simulation centre should become a part thereof and contribute to the interlinking of risk and quality management. 

**Cooperation of hospitals: **One interview partner stated that not every hospital needed its own simulation centre. Especially larger hospital networks could establish a shared simulation centre which could be used by all cooperating hospitals. This form of cooperative share of resources appears also feasible for hospitals in metropolitan areas. 

## Discussion

The WHO “Patient Safety Curriculum Guide” alone mentioned the word “simulation” 37 times (no titles or sources) [16]. Still, it is a long way to go from the WHO’s high-level implicitness to the de facto establishing of simulation centres in everyday hospital life. We interviewed heads of departments, researchers, and hospital executives regarding organisational and economic aspects. 

Our survey disclosed how divergent the arguments were regarding the establishment of simulation centres. Considering the relatively small size of the community in this field this is somewhat a surprise since one would expect a more coherent chain of arguments amongst the supporters. 

Additionally surprising was the fact that the interviews gave no evidence that the use or success of simulation centres were being monitored systematically. The arguments were kept very superficial along the lines of very abstract levels such as contribution to patient safety, optimizing of vocational training, increase in employee satisfaction, or improvement of hospital’s reputation.

So far there are only very few surveys on the economic profitability of simulation centres on hand – we know of none in the German health care sector. One US publication calculated that the training of surgical residents in the operating room causes a significant increase in operative time that justifies and necessitates alternative forms of trainings and preparation for residents [17]. Another US study compared the initial cost of establishing a simulation centre for the application of central venous catheters with later cost savings [14]. It showed that the cost reduction due to lowered infection rates and lowered duration of stay at an ICU surpassed the training costs sevenfold.

To our knowledge this study was the first survey with an emphasis on hospital executives and their motivation and argumentation for the implementation of simulation centres.

### Simulation centres as part of risk and quality management system

The interviews indicated that the interplay of quality and risk management and the simulation centres could be improved. So far hardly any conclusions were drawn from CIRS-reports to be incorporated in simulation trainings and to be used to avoid causes of errors in the future according to our interview partners. 

At least one institution considered the organisational approach of simulation trainings as a means of a superior risk and quality management. This could set a trend: when simulation trainings are seen holistically as a part of a structured clinical quality and risk management they could be used to test and improve processes, document their consequent implementation, but also to avoid errors. This could help meet the increasing demands of quality- and risk management in health care legislation such as minimum standards for risk management and mandatory error reporting systems in hospitals.

#### Focus and measuring of success of simulation centres

Some of the interview partners criticized simulator trainings‘ common lack of focus and objective. It seems there was no apparent systematic differentiation of skill training (focus on the acquisition of technical skills) and team trainings (focus on social factors such as communication and effective team work). A lack of formulated objectives and didactic focus could reduce the efficacy of trainings.

Apart from learning objectives the economic benefit of simulation training and simulation centres in hospitals should be defined and tracked explicitly and measurably. The benefits could be measured on various levels: decreasing error figures can have positive effects on profitability; a decline in liability cases due to decreasing error risks could keep insurance rates low. In the long run simulation centres will have to pay off – like any other business unit of hospitals – in order to continue operating successfully. A prerequisite to do so is a system of figures that can measure, monitor and compare the effects of simulation trainings. The survey on hand shows an immense backlog on that part. 

#### Suggestion for a KPI system

**Figure 1:**
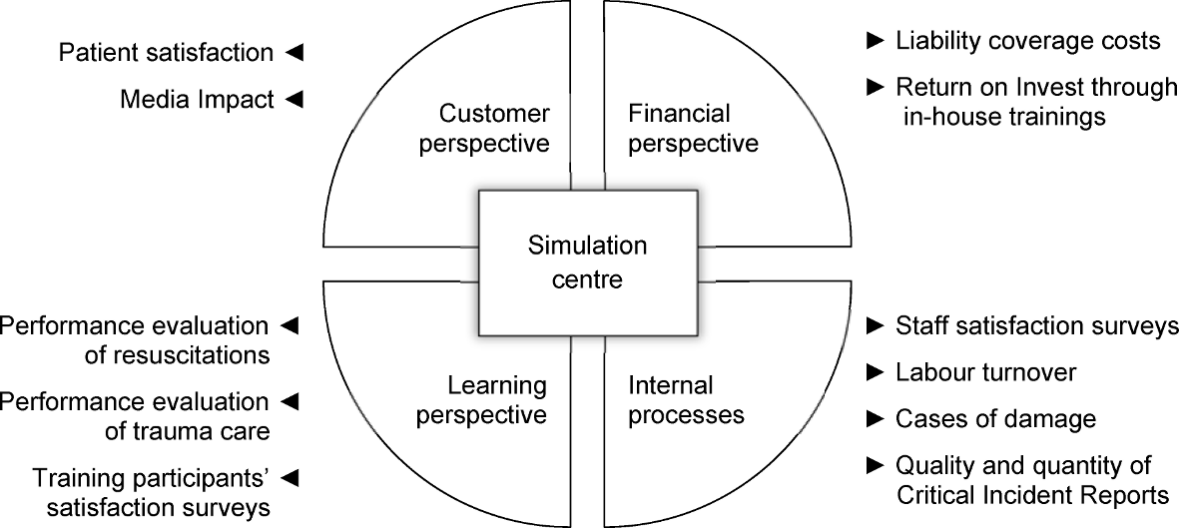
KPI system for monitoring the impact of a simulation centre in hospitals. The indicators are structured along the four dimensions of the Balanced Scorecard System.

None of the interview partners reported an existing, comprehensive KPI system monitoring the impact of the simulation centre in their institutions. Nevertheless, they mentioned some individual indicators. Based on these, we suggest a combination of several indicators to describe and monitor the purpose and impact of a simulation centre on organisational level. See figure 1 (Fig. 1) for an outline of possible performance indicators based on our interviews. The items have been grouped according to the dimensions of the Balanced Scorecard (BSC) which is a common concept in business economics [18]. A BSC does not only comprise financial aspects but offers a holistic perspective on an organisation´s strategic development, like the internal business processes, the learning and development of the organisation, and the customer perspective. It aims to provide decision makers with an understanding of their organisation that allows decision-making for long term interests. 

#### Limitation and outlook

Many aspects in the borderland of medicine and business administration are unexplored. The distinct explorative approach of this study and the selection of few expert participants enabled insight in implementation argumentations and considerations of profitability for simulation centres in German hospitals. Due to the small number of participating interview partners the survey is not representative.

The small sample group size results from the nature of the German simulation community as well as from the design of this study itself. Having little pre-existing research regarding business aspects of simulation centres available to start from, a qualitative and exploratory study design was chosen to open this field.

Whilst there seems to be a rising number of offers from commercial simulation training companies for the medical sector, we wanted to specifically address those hospitals operating an own simulation centre in-house. The interview partners have been identified by researching publications, internet searches and screening of congress programmes. Whilst this implies a fair chance to identify most of the supposed relevant interview partners in this field, it still bears the risk that some hospitals were not detected that are running an own simulation centre but do not contribute back to professional public by publication of research.

Given the high-profile interview partners who mostly had managing responsibility, the response rate of 11 out of 24 requests seems acceptable, bearing in mind that the interview took up to 45 minutes which is a significant time effort for a senior leadership position. 

For an exploratory study design with such diversity in the sample group we consider the low number of participants acceptable, especially as they cover all areas of interest for the qualitative survey. Future research can build on these results and focus more on one of the identified aspects. This will also allow to optimise the study design towards individual sample groups e.g. through specific questionnaires. Also, the outlined set of KPIs must be challenged and founded on practical experiences.

The exact analysis of profitability of simulation trainings and centres in hospitals with respect to manifold influencing factors is a commendable starting point for future interdisciplinary research. The same applies to a closer analysis of clinical risk management and approaches to combine it more effectively with quality management. To evaluate and steer all these organisational and economic aspects a system of economic indicators should be developed without which an assessment of benefits and progress seems near impossible.

## Competing interests

The authors declare that they have no competing interests. 

## Note

The authors Tobias Rampel and Benedict Gross contributed equally to this paper in sense of a joint first authorship.

## Supplementary Material

Interview Guideline

## Figures and Tables

**Table 1:**
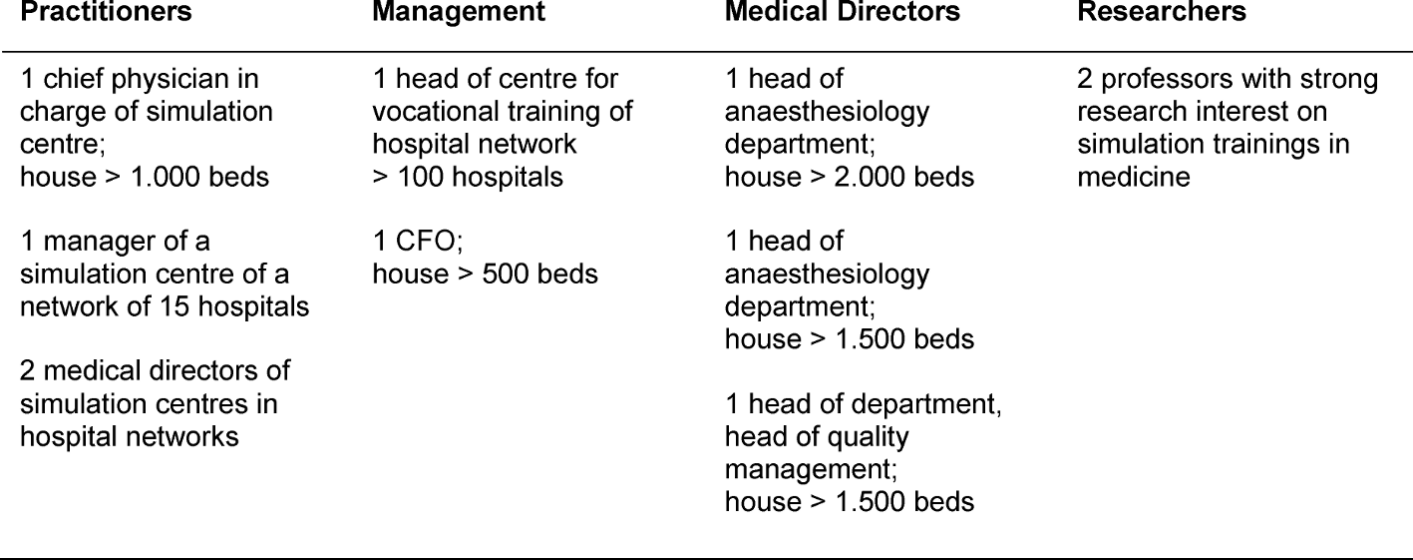
Participants of the survey
