# Supplementary material for: Simulation centres in German hospitals and their organisational aspects: Expert survey on drivers and obstacles
Source: GMS J Med Educ. 2018 Aug 15;35(3):Doc40. doi: 10.3205/zma001186 (PMC6120149; doi:10.3205/zma001186)
Supplement: Interview Guideline [file JME-35-40-s-001.pdf]

Simulation centres in German hospitals and their organisational aspects: Expert survey on drivers and obstacles. GMS J Med Educ. 2018;35(3):Doc40.

Attachment: Interview Guideline

Introduction

- Name and background of the interviewer
- Purpose of the study
- Explain confidentiality and ask for consent of the interviewee

General Questions

1. Please describe type, length and scope of the simulation trainings offered in your institution.
2. Detailed questions for every training format mentioned:
   a) Since when have you been offering these training?
   b) How many employees have been participating this training until today?
   c) What are the scheduled intervals of these trainings?

Motivation for Implementation

Thank you for the information so far. Now, please think back to the implementation-phase of simulation trainings and the organisational environment.

3. What reasons argued for the implementation of simulation trainings?
4. Which economical aspects were crucial?

Financing

5. How are simulations trainings financed?
6. How are simulations trainings calculated?
7. Who is in charge of budgeting and how is the budget planned?
8. How do you reason with (commercial) management if you need grants for simulation trainings?

Questions regarding possible Key Performance Indicators (KPI´s)

Thank you again for this information. We are almost at the end of my questions.

9. Are there any indicators to measure the impact of simulation trainings? Examples: satisfaction, performance.
10. Have you done an analysis of key figures before and after the implementation of simulation trainings? Examples: labour turnover rate, error frequency.
    a) Which key figures did you analyse?
    b) How did they change over time?
11. Risk management systems and Critical Incident Reporting Systems (CIRS) are now required by German law. How are simulation trainings connected to
    a) the mentioned systems?
    b) the quality management system?
    c) Clinical Governance for patient safety?
    d) any other?
    e) How is related data used - if collected?

Conclusion

We have come to the end of the interview.

12. Is there anything else regarding the topic of this interview we did not touch?
